# Rare pediatric Galeazzi lesion with progressive wrist deformity

**DOI:** 10.1080/23320885.2026.2638084

**Published:** 2026-03-03

**Authors:** Sarah Kerler, Johannes Fuchs, Jörg Grünert, Samuel Christen

**Affiliations:** ^a^ Department of Hand and Peripheral Nerve Surgery, Berit Klinik, Goldach/St. Gallen, Switzerland; ^b^ Children’s Hospital of Eastern Switzerland, St. Gallen, Switzerland

**Keywords:** Galeazzi-equivalent, Galeazzi fracture, pediatric forearm fracture, DRUJ

## Abstract

Galeazzi injuries are rare in early childhood and may be missed at initial presentation. Undiagnosed instability of the distal radioulnar joint can lead to progressive deformity and functional impairment. We report a rare case of Galeazzi injury in early childhood that led to progressive wrist malalignment during growth. Over the years this resulted in a severe restriction of forearm rotation. Using computed tomography–based three-dimensional analysis, a multiplanar corrective osteotomy was planned with reference to the contralateral healthy side. The procedure reduced the initial supination deficit of 120° to 20° and restored excellent wrist function. Missed Galeazzi injuries in early childhood can result in progressive deformity and significant long-term functional limitations. Advanced three-dimensional imaging enables accurate analysis of complex deformities and facilitates corrective osteotomy. Even in delayed presentations, satisfactory functional recovery can be achieved with appropriate surgical planning.

## GRAPHICAL ABSTRACT

Algorithm for the assessment of the distal radioulnar joint (DRUJ) in severe distal forearm fractures. CRIF = closed reduction and internal fixation.

**Figure:**
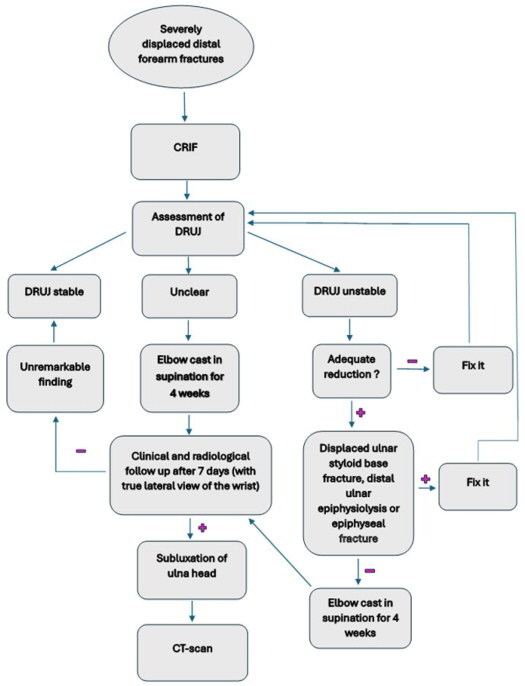

## Introduction

In 1934, the Italian surgeon Riccardo Galeazzi first described, based on 18 cases, the biomechanics and pathology of a combined injury involving a distal third radial shaft fracture with simultaneous (typically dorsal) dislocation of the distal radioulnar joint (DRUJ) [1,2]. While the incidence of Galeazzi fractures in adults accounts for approximately 7% of all distal forearm fractures, these injuries are considerably rare in children, occurring in only 0.3–2.8% of the cases [3–6]. The relatively high elasticity and tensile strength of ligamentous structures generally protect the pediatric wrist from dislocations in general. The so-called Galeazzi-equivalent injury, which combines a distal radius fracture with severely displaced distal ulnar epiphysiolysis, is slightly more common in children [2,6–8]. Ulnar dislocation with distal metaphyseal fractures of both bones is extremely rare and reported only in isolated pediatric cases [5,9,10].

## Patients/materials and methods

A 12-year-old boy presented to our pediatric hand surgery clinic with progressive restriction of forearm rotation of his left wrist following an injury in early childhood.

**Figure 1.**
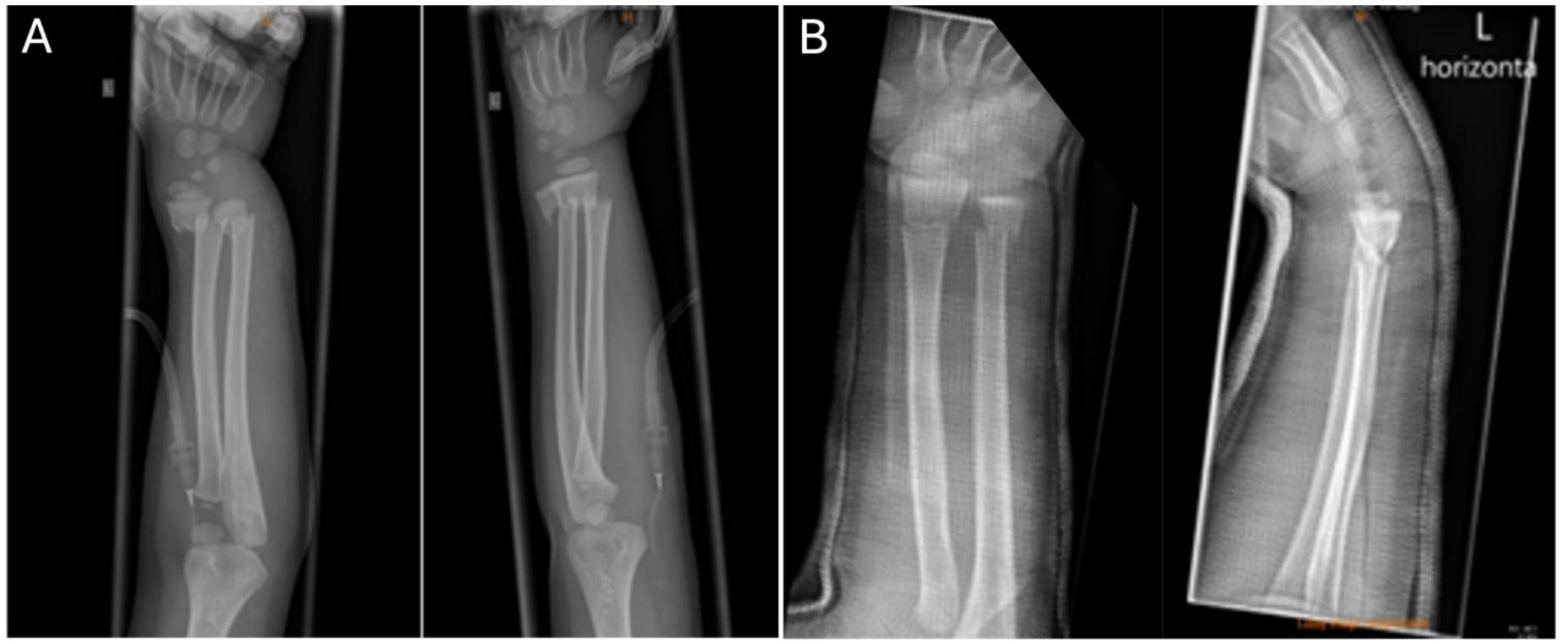
X-rays before (A) and after (B) closed reduction of the fully displaced forearm fracture.

**Figure 2.**
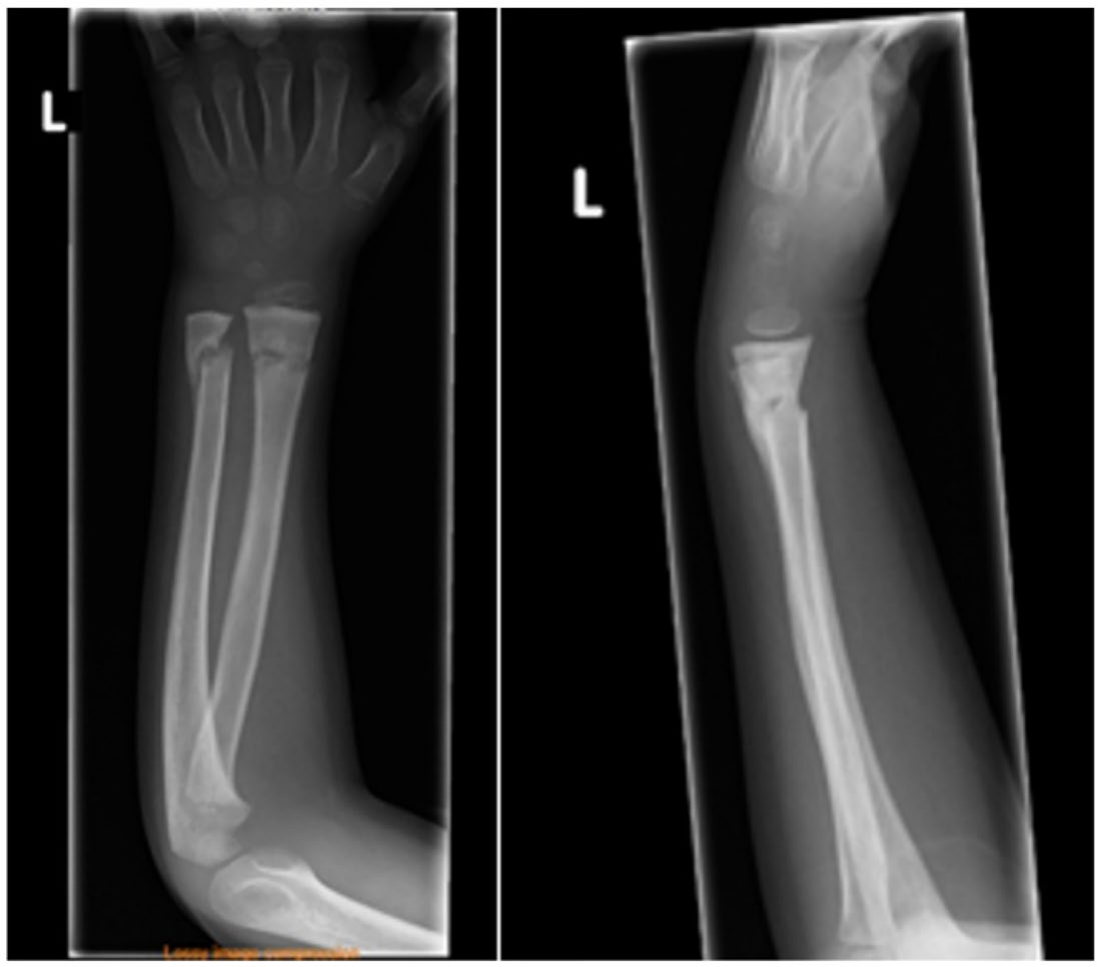
X-ray follow-up 4 weeks after trauma.

At the age of 4, he suffered a severely displaced distal forearm fracture of the left arm after a fall from 3 meters (Figure 1). The fracture was initially managed at an external hospital with closed reduction and immobilization in an elbow cast for 4 weeks, followed by a forearm cast for an additional 2 weeks. Radiological follow-up at 4 weeks demonstrated only a slight dorsal tilt of the ulna (Figure 2).

**Figure 3.**
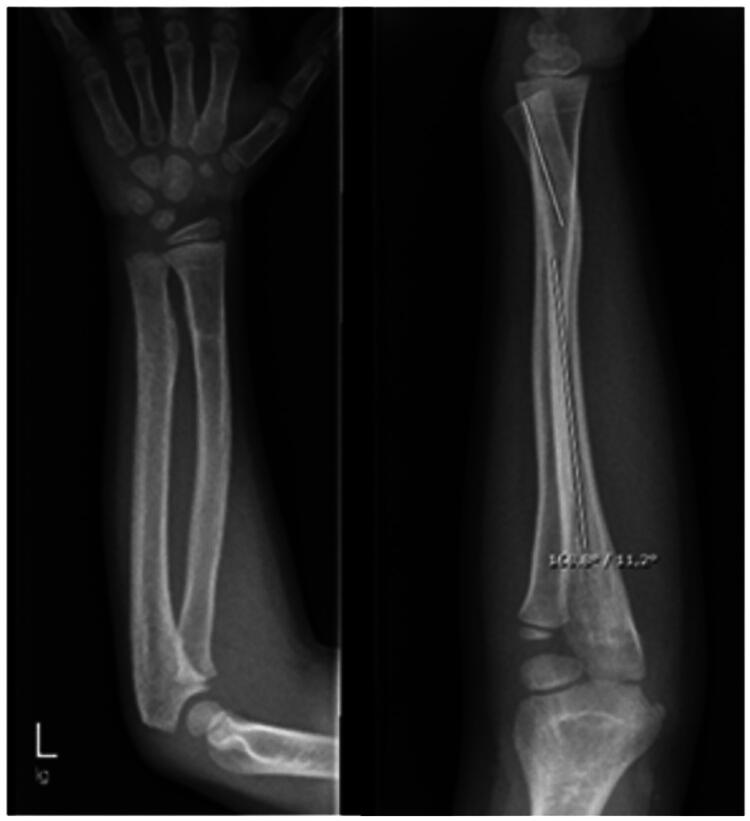
2-year follow-up showing a progressive dorsal angulation and subluxation of the distal ulna.

At 24-month follow-up, a supination deficit of 45° was documented, while pronation remained unrestricted. Conventional radiographs revealed progressive dorsal angulation of the ulna with subluxation of the distal radioulnar joint (DRUJ) (Figure 3). As the patient was asymptomatic and spontaneous correction during growth was expected, a watchful waiting strategy was adopted.

**Figure 4.**
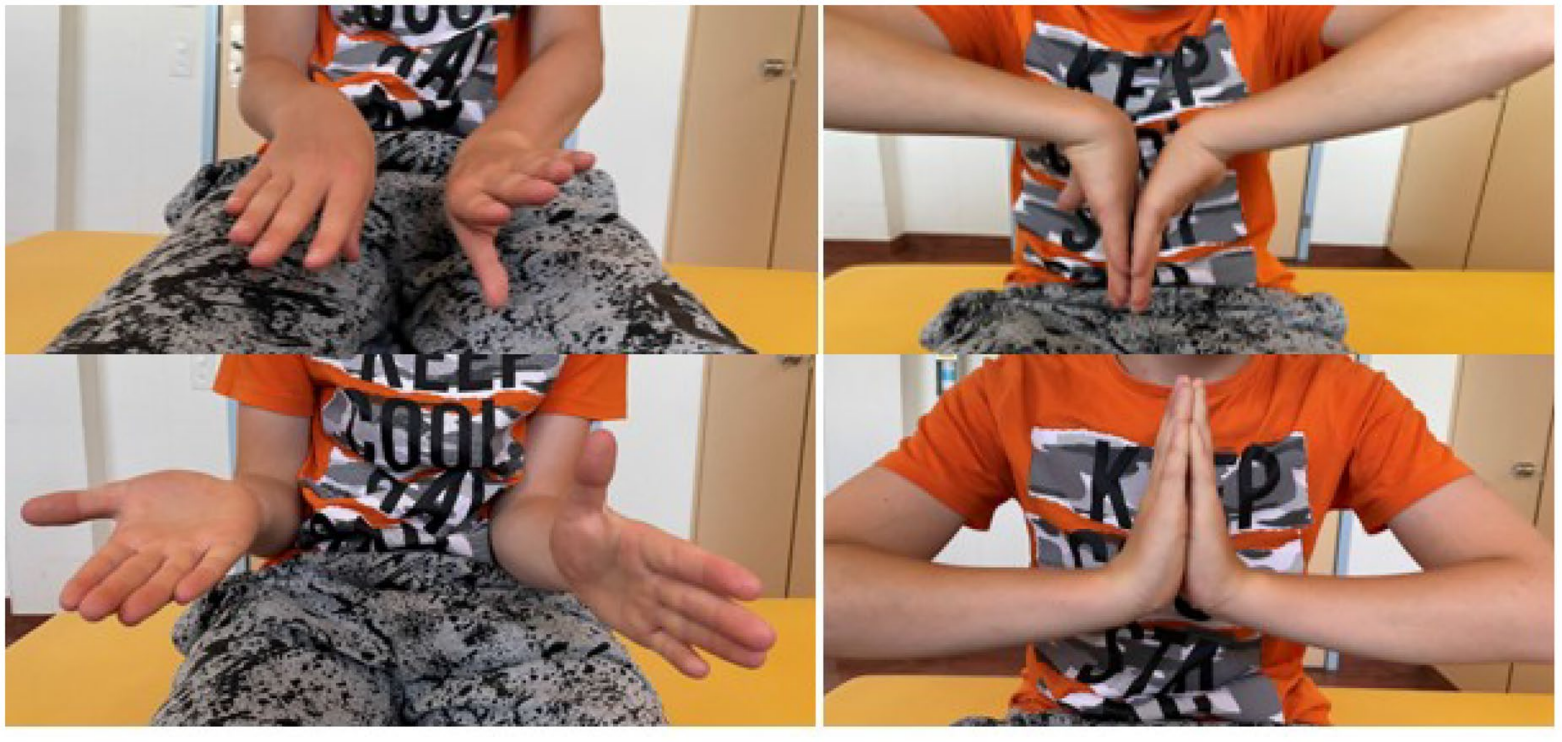
Clinical finding at initial presentation in our outpatient clinic 8 years after trauma. Range of motion: Pronation-Supination 90°-0°-90° (right) vs. 110°–30°–0° (left), Flexion-Extension 80°-0°-80° (right) vs. 60°-0°-80° (left).

**Figure 5.**
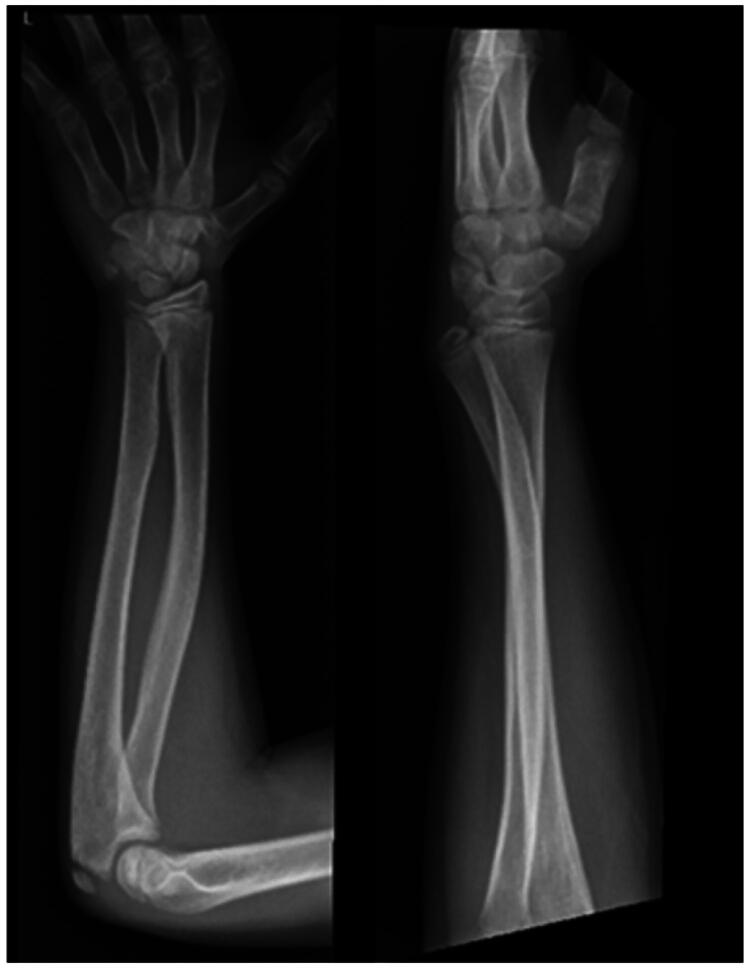
X-ray 8 years after injury showing marked angulation of both forearm bones and dorsal dislocation of the ulna in the DRUJ.

Over the years, the motion restriction progressively worsened. Due to increasing limitations in daily activities and the patient’s desire to pursue a manual profession, he presented to our pediatric hand surgery clinic for a second opinion. Wrist supination was now limited to −30° (total deficit of 120°), while at the same time a hyperpronation of +20° could be observed. Furthermore, wrist flexion was restricted by 20° (Figure 4). Conventional radiographs revealed marked dorsal angulation of the distal ulna with DRUJ dislocation (Figure 5).

**Figure 6.**
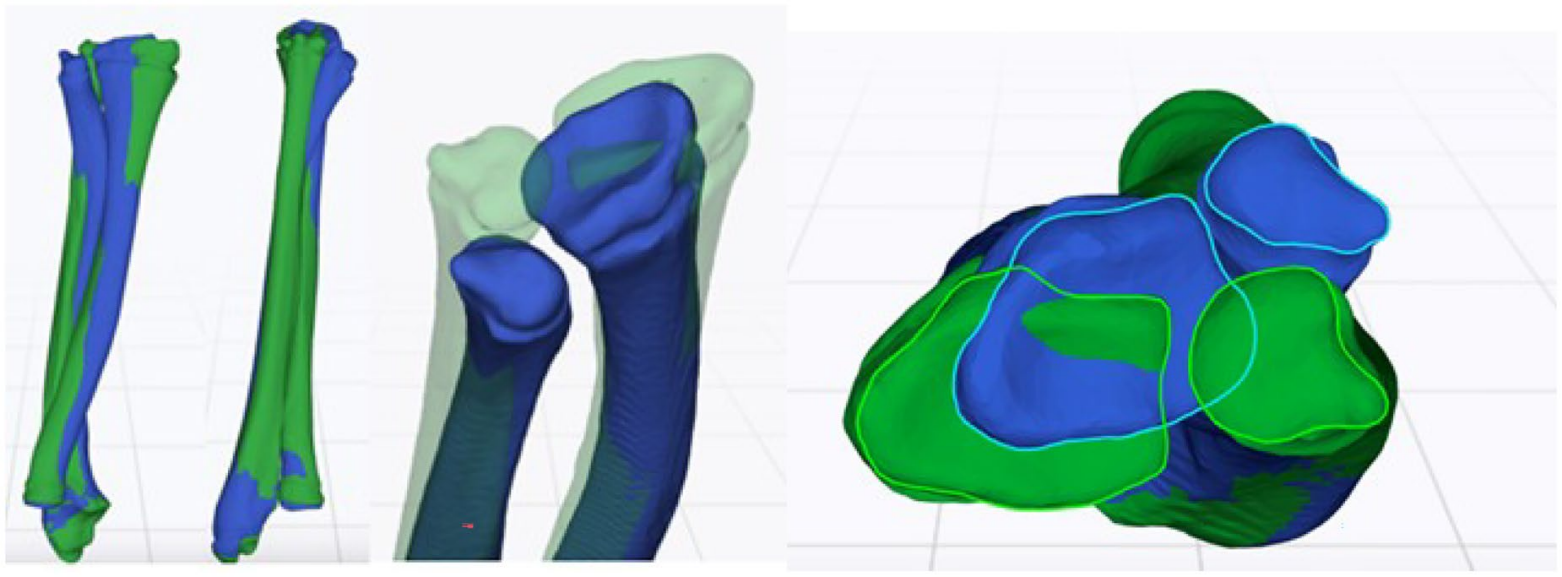
Three-dimensional analysis and surgical planning (Medartis AG, Basel, Switzerland), blue: affected side, green: mirrored contralateral side.

**Figure 7.**
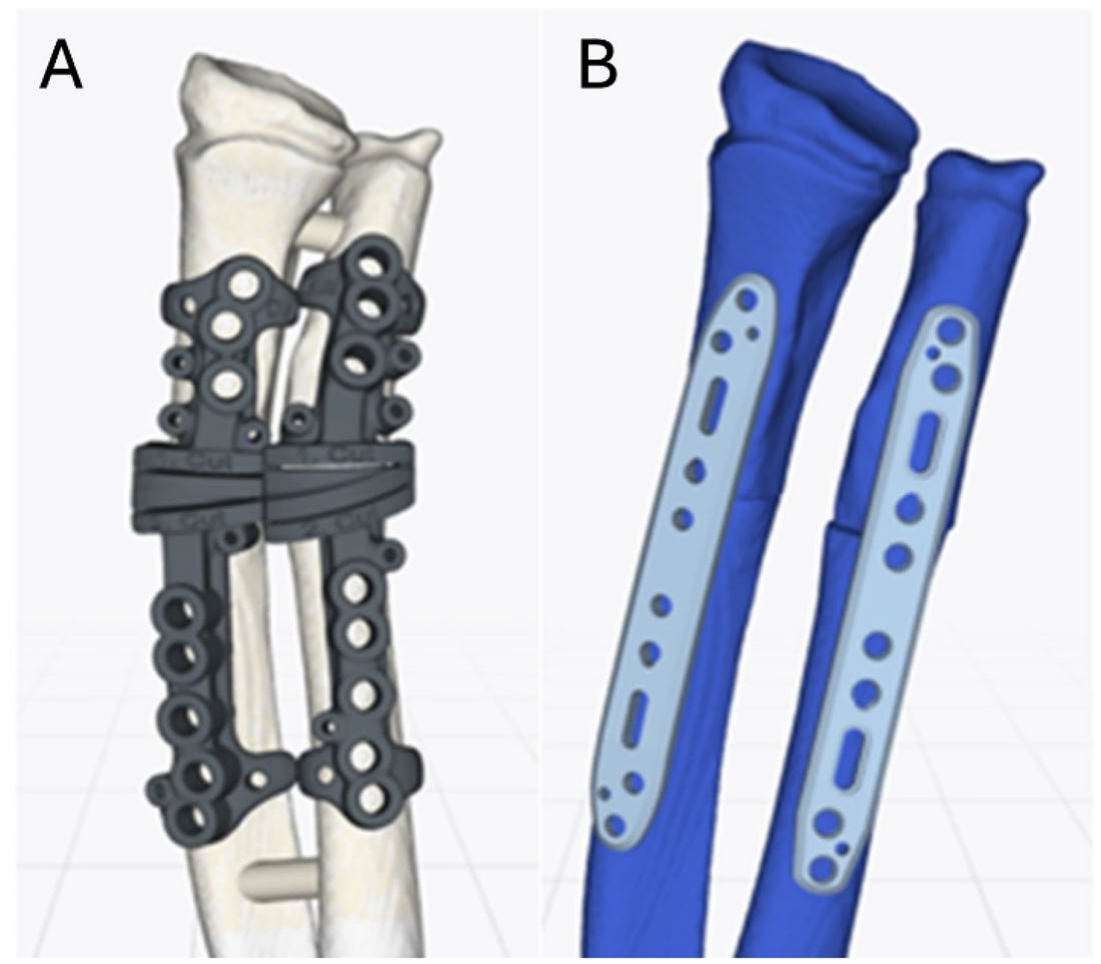
Preoperative planning of patient-specific drilling and cutting guides (A), and osteosynthesis with standard plate fixation in corrected alignment (B).

Comparative 3D analysis using computed tomography of both forearms revealed multidirectional deformities of the distal third of both radius and ulna (Figure 6). A multiplanar corrective osteotomy of both bones was planned using the CMX system (Medartis AG, Basel, Switzerland) with patient-specific surgical guides (Figure 7).

## Surgical technique

**Figure 8.**
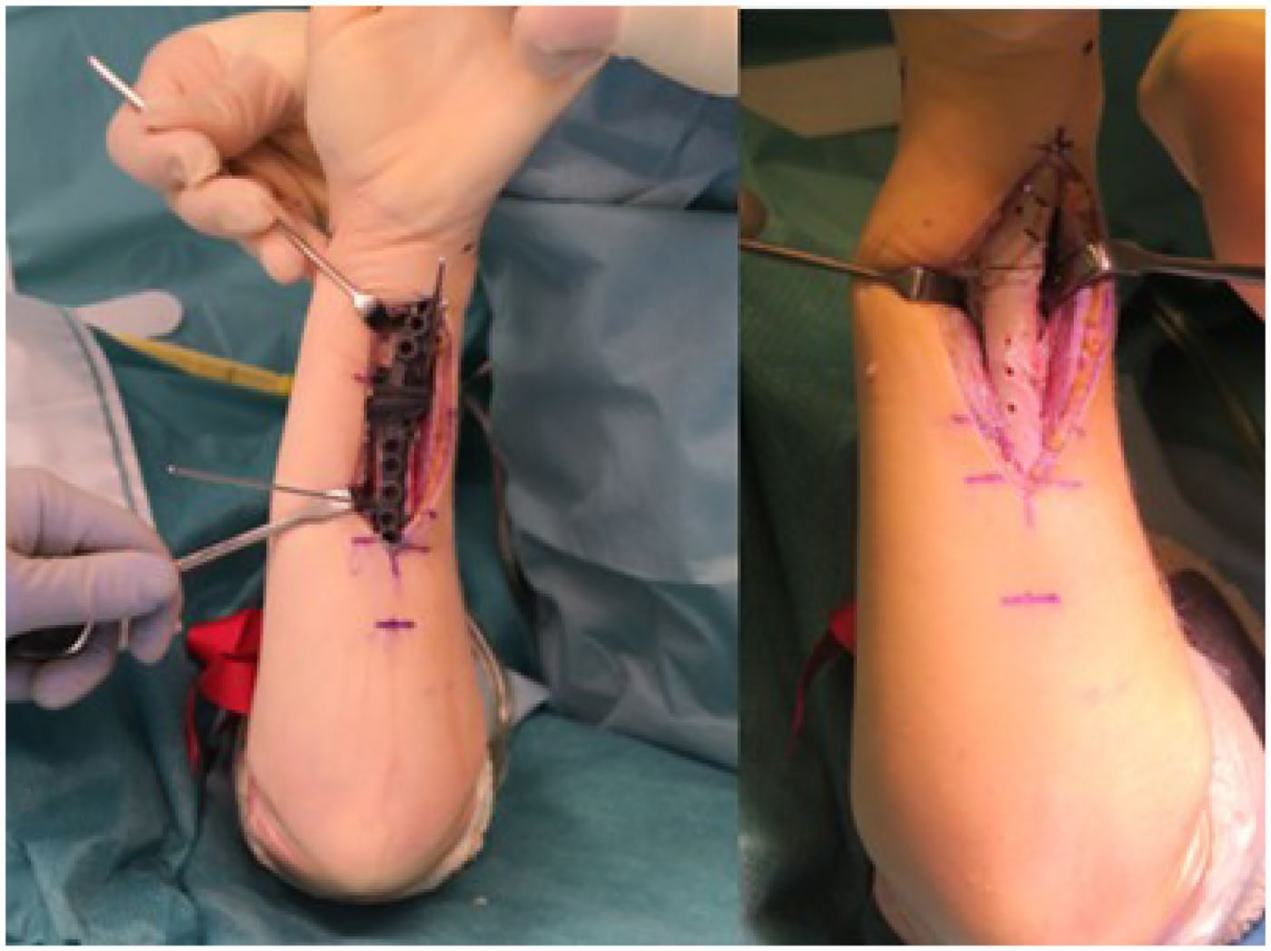
Guide positioning on the ulna using K-wire fixation (A). Predrilled screw holes and wedge osteotomy after removal of the guide (B).

Given the inability to supinate the forearm, the ulnar osteotomy was performed as the first step after predrilling the screw holes according to the guide (Figure 8). Afterwards the forearm could be repositioned to allow access to the distal radius *via* a modified Henry approach. For guide placement, the flexor pollicis longus muscle had to be detached from the radial shaft.

**Figure 9.**
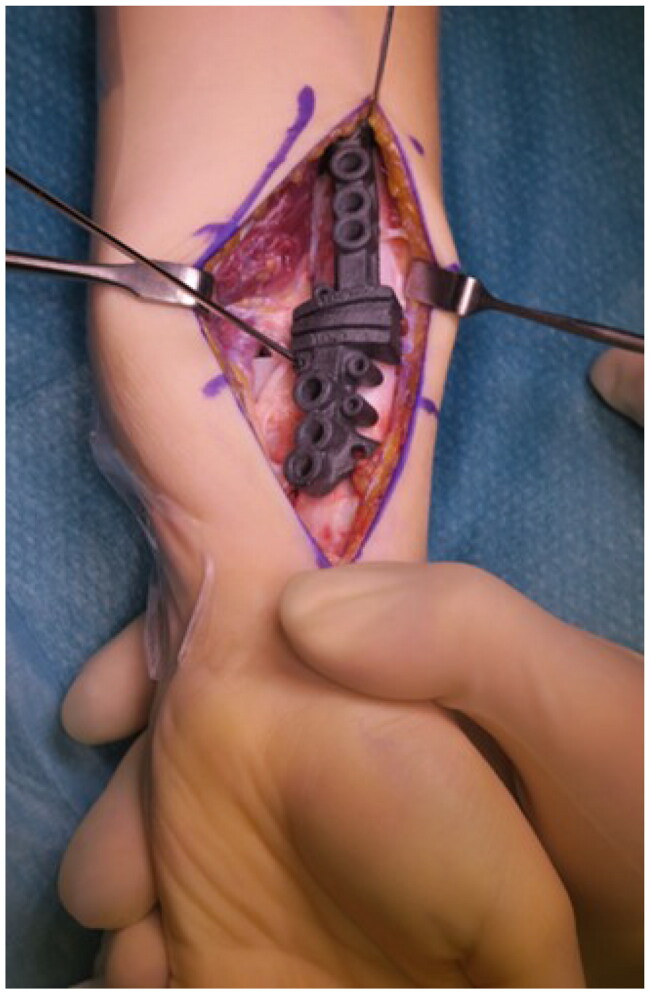
Positioning and temporary fixation of the radial guide after exposure of the bony references.

Following the wedge osteotomy of the radius both bones were reduced and osteosynthesized with standard forearm shaft plates (Radius Ulna Shaft System 2.8, Medartis AG, Basel, Switzerland). The reduction was obtained by means of the screw hole placement defined by the guide, consistent with the preoperative planning (Figures 7, 8, 9).

**Figure 10.**
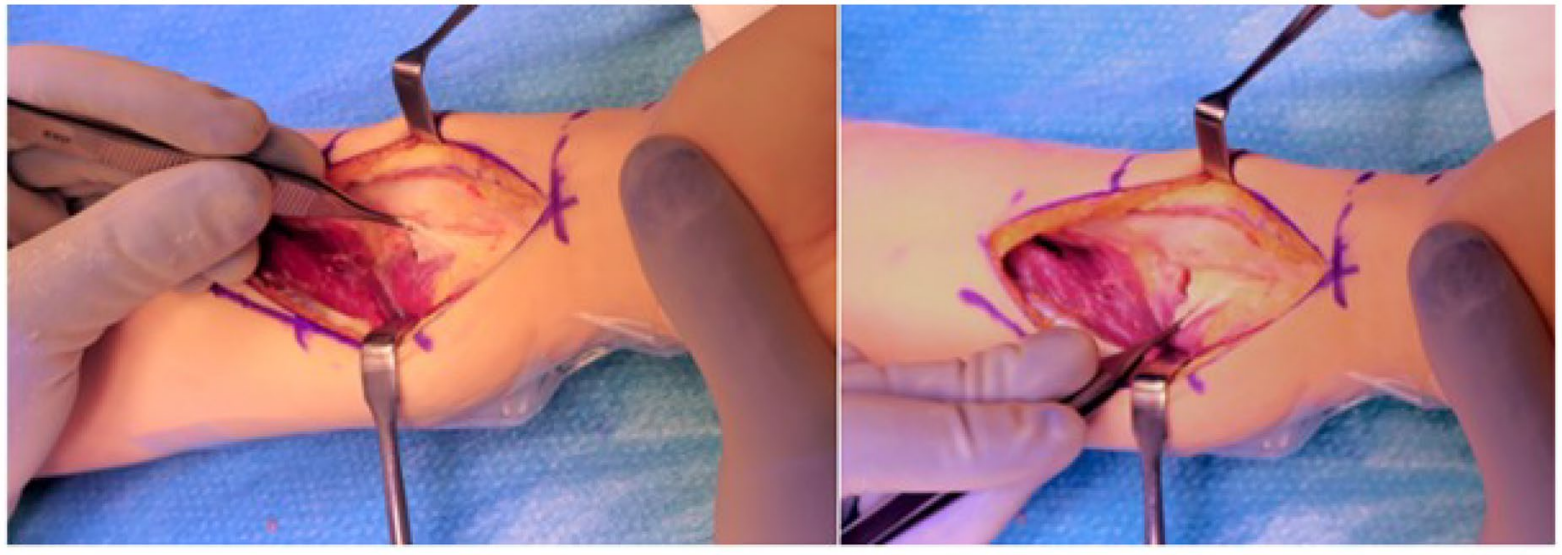
Pronounced thick scarring of the distal interosseous membrane and the pronator quadratus muscle residue.

**Figure 11.**
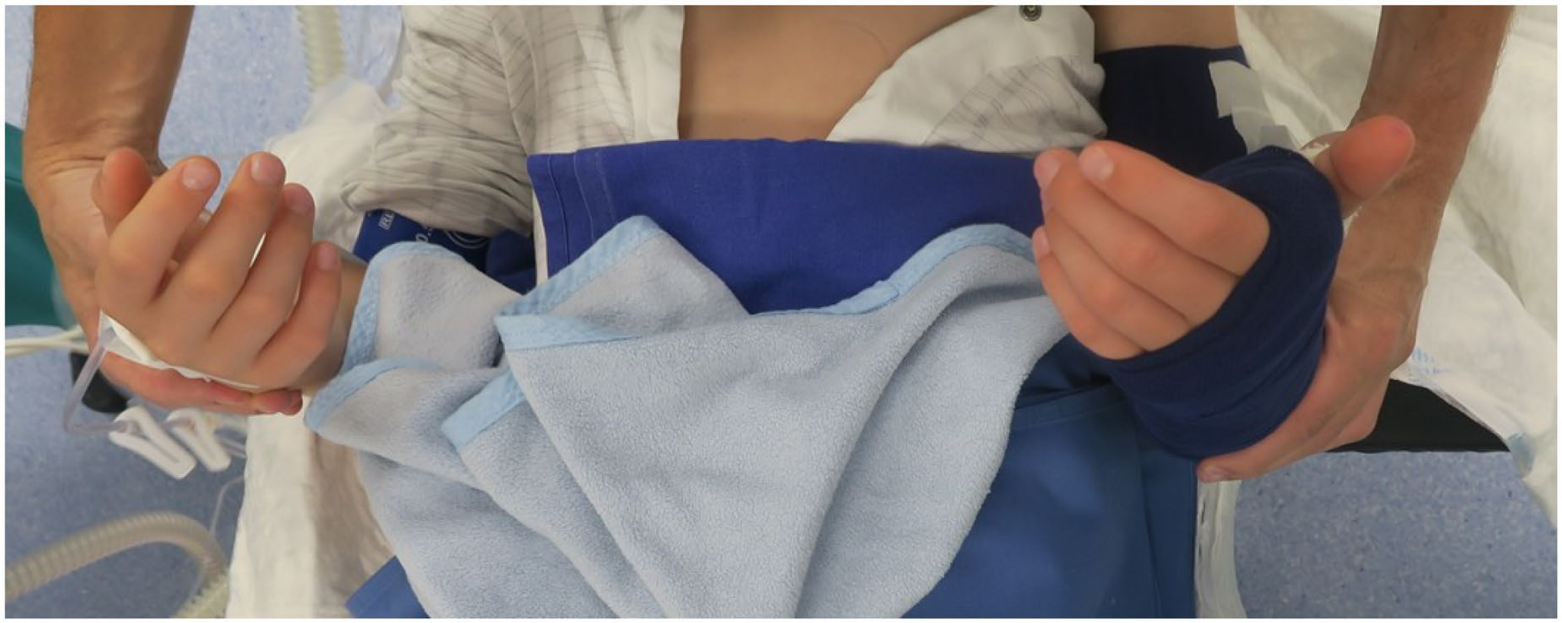
Postoperative result showing an achieved supination of 40° (in redressing cast).

After bony correction, supination remained limited to 0°, prompting an additional soft-tissue release. In the distal third of the shaft, a thick fibrous scar formation of the interosseous membrane could be identified. In addition, the pronator quadratus muscle had been replaced by scarred residue (Figure 10). After excision of the scar tissue and further soft-tissue release, supination improved to +40° (Figure 11). An above-elbow plaster splint was applied with the forearm positioned in maximal supination.

## Figures and Tables

**Figure 12.**
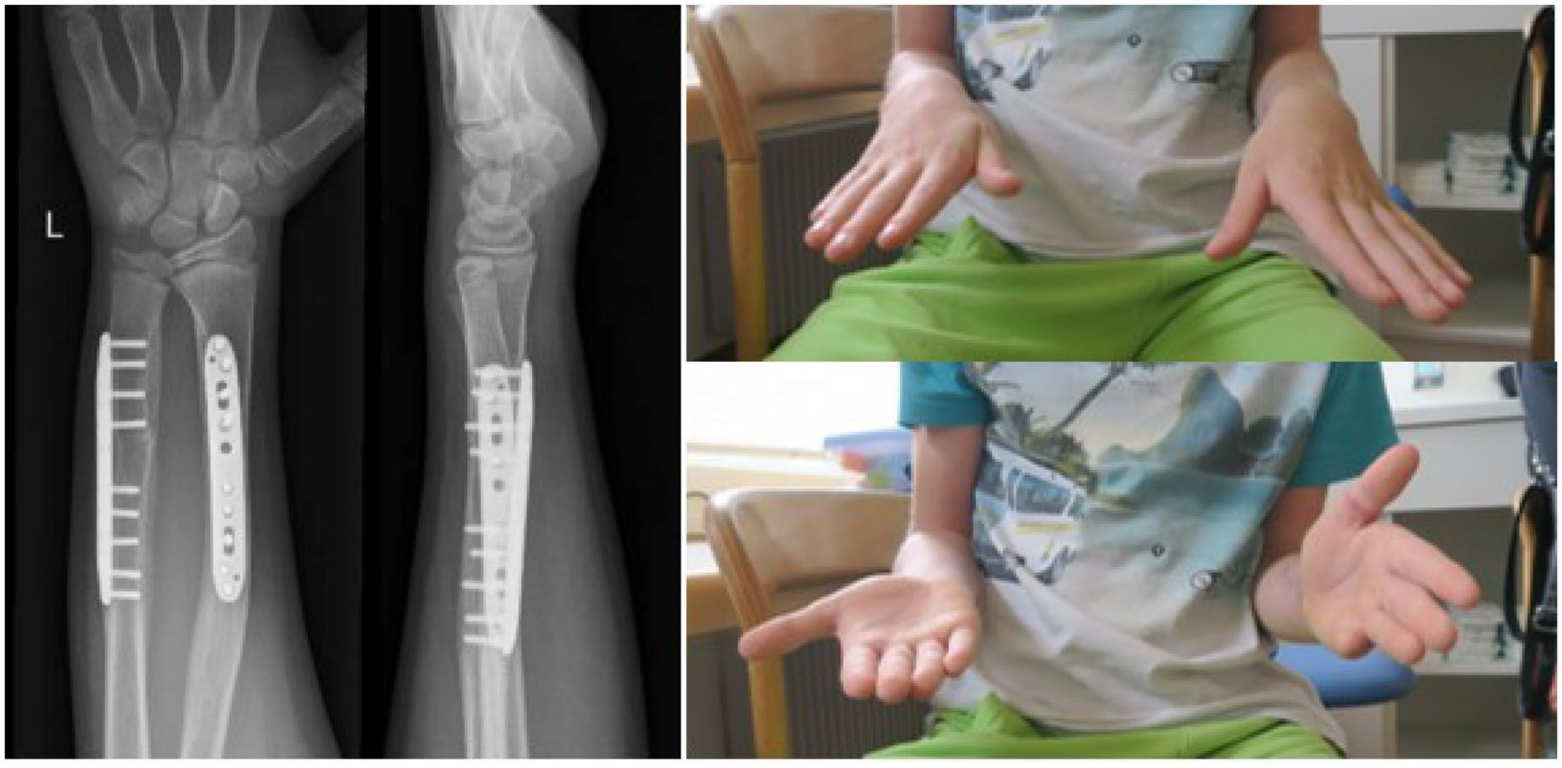
Clinical and radiologic finding 3 months after corrective surgery. Forearm rotation of left side: Pronation-Supination: 70°-0°-0°.

**Figure 13.**
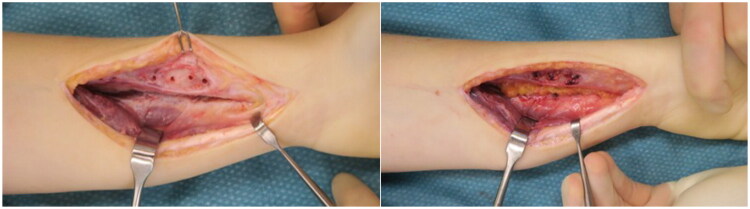
Revision surgery 1 year after corrective osteotomy with hardware removal, extensive soft tissue release and fat graft interposition.

**Figure 14.**
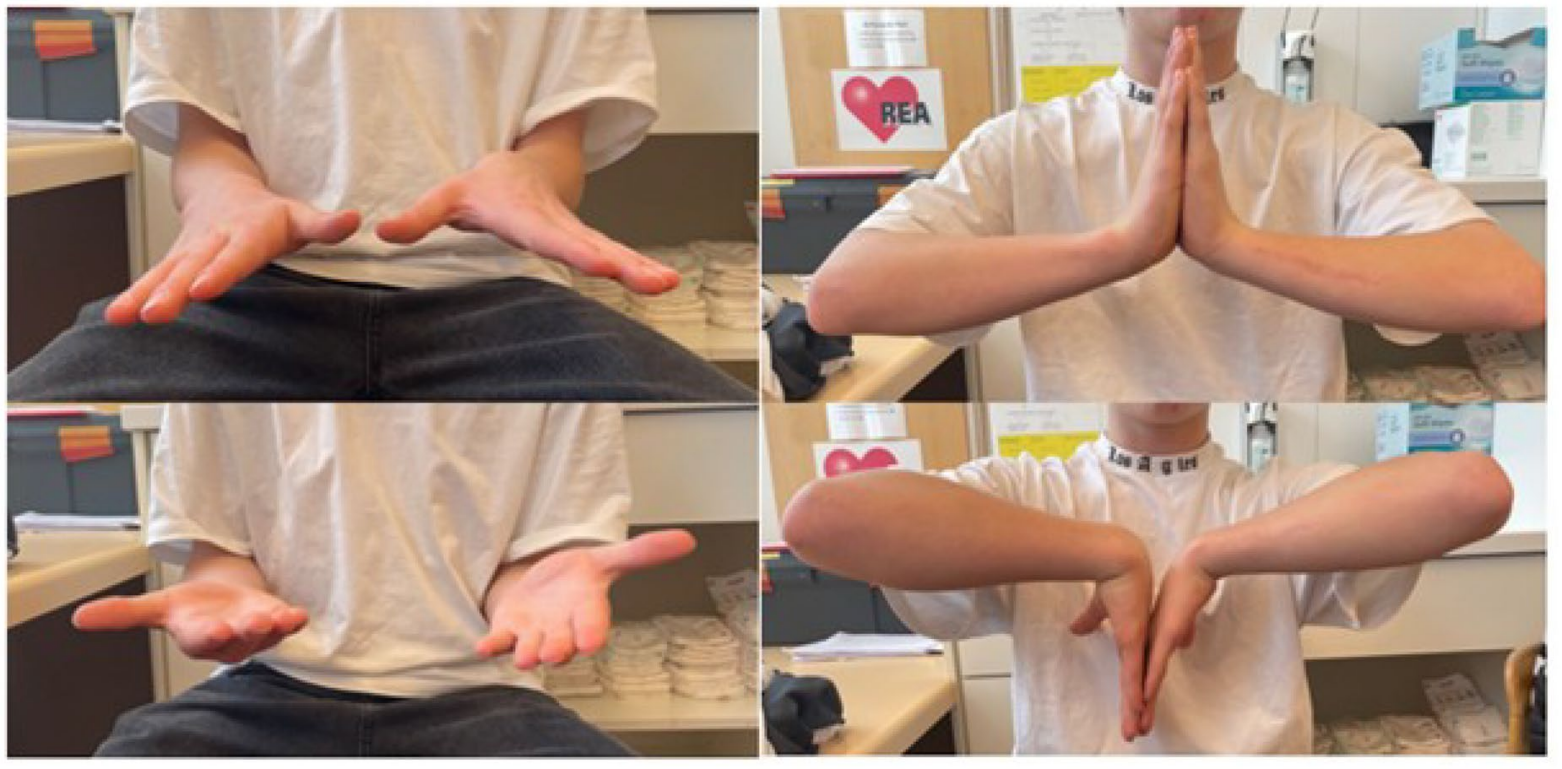
1 year after hardware removal documenting an almost normal range of motion of the left forearm/wrist. Range of Motion of left side: Pronation-Supination 80°-0°-60°, Flexion-Extension 70°-0°-90°.

## Results

The initial postoperative course was uneventful. However, despite early functional rehabilitation, the range of motion progressively deteriorated, partly due to temporary poor patient compliance. Three months postoperatively, supination was limited to 0° again (Figure 12). Inadaquate bony correction was excluded by repeat three-dimensional analysis.

Supination did not improve further despite intensive occupational therapy. Therefore, we decided to remove the osteosynthesis material and perform another soft tissue release twelve months after the corrective osteotomy. The severely re-scarred interosseous membrane was extensively released and subtotally resected in its distal portion. In addition, a arthrolysis of the DRUJ was performed by releasing the palmar joint capsule. Supination subsequently improved to 70°. To prevent recurrent scarring, a fat tissue graft was interposed between the radius and ulna (Figure 13). This time, immediate intensive mobilization was initiated under occupational therapy guidance and continous plexus catheter analgesia in an inpatient setting.

At final follow-up, one year after hardware removal, supination of 70° could preserved, leaving the patient unrestricted in daily activities and during his apprenticeship as car mechanic (Figure 14).

## Discussion

We report a case of a distal forearm fracture in a young child, in which an initially subtle distal radioulnar subluxation progressed over time to complete dislocation with increasing malalignment of the wrist. To our knowledge, no comparable case has been described in the literature so far.

The Galeazzi-equivalent injury is defined as the combination of a pediatric distal radius fracture, typically displaced palmarly, with a markedly dislocated distal ulnar epiphysiolysis corresponding to a Salter–Harris type I or II lesion. However, rare variants of Galeazzi-equivalent fractures have been described in the literature, including splitting of the ulnar epiphysis consistent with Salter–Harris type III or IV injuries. In two case reports [11,12], the development of a Y-shaped ulnar bifurcation was reported as a long-term complication following missed diagnosis of such an injury.

In his original description, Galeazzi postulated that in fractures of the radius associated with dislocation of the ulnar head, the distal radial fragment is typically pronated and drawn toward the ulna by the muscular forces of the pronator quadratus, adductor pollicis longus, and extensor pollicis brevis. These muscles exert a spiral force around the fragment, resulting in palmar displacement of the distal radial fragment. Owing to these muscular forces, dislocation of the DRUJ can also occur gradually during the subsequent course [1,2]. Awareness of the potential for delayed displacement is particularly important when a conservative treatment strategy is pursued.

In the presented case, intraoperative examination revealed a markedly scarred pronator quadratus muscle. We propose that this scarring resulted in a progressive contracture, which, over time and with continued growth, contributed to progressive secondary displacement.

Assessment of DRUJ stability in children, particularly in acute trauma and toddlers, remains challenging [5]. The incidence of DRUJ instabilities associated with wrist injuries is not known. It is assumed that there is a certain degree of underreporting [4,5,7,9,13]. Clinically, a prominent or, less commonly, depressed ulnar head in comparison to the contralateral side, increased DRUJ mobility, and localized tenderness over the distal radioulnar region may indicate DRUJ injury [3,7].

Imaging assessment of the DRUJ in pediatric patients appears unreliable: The ossification center of the distal ulna typically appears only between 5 and 7 years of age, rendering the ulnar head invisible on conventional radiographs in younger children [6,14]. Epiphysiolysis or avulsion injuries of the ulnar styloid process may also be undetectable on X-rays at this age. In addition, optimal radiographic projections are often difficult to obtain due to limited patient compliance. Although modern CT imaging has reduced radiation exposure, concerns regarding its use in children remain [6,15]. MRI typically requires general anesthesia in preschool-aged children, limiting its routine use.

Distal radioulnar joint (DRUJ) instability in children is exceedingly rare. DRUJ instability in the pediatric population occurs only in the context of severely displaced forearm fractures requiring surgical intervention. Therefore, assessment of DRUJ stability should always be performed intraoperatively and during postoperative follow-up in such injuries, although this evaluation may be challenging. The graphical abstract proposes an algorithm for the management of these cases.

In contrast to adults, conservative treatment of Galeazzi fractures is generally possible in children. While non-operative management of classic Galeazzi fractures in adults yields unsatisfactory results in over 90% of cases [5,9], children almost invariably achieve good to excellent outcomes [3–5,7,16]. Closed reduction followed by immobilization with an above-elbow cast in supination for 4–6 weeks remains the gold standard [3–5,9]. Surgical intervention is rarely required; when performed, minimally invasive osteosynthesis of the fracture with closed reduction and fixation using Kirschner wires or intramedullary nailing is usually sufficient [5,9,16–18]. Surgical stabilization of the DRUJ is generally not necessary [5].

In our case, post-reduction radiographs retrospectively revealed a subtle ulnar subluxation (Figure 3). Over time, the malalignment of the ulna increased. It is plausible that these forces contributed to the progression of the initially minor subluxation in our case. The progressive DRUJ dislocation in this patient also appears responsible for the unusual progressive angulation of the distal forearm during growth. In general, malalignments—especially those of metaphyseal fractures located near a potent growth plate such as at the distal radius—are reliably remodeled during growth [14].

Persistent or increasing functional limitations after pediatric fractures warrant further evaluation. Forearm rotation restrictions, as observed in this case, may be partially compensated by the highly mobile shoulder joint. Children can effectively compensate such deficits, which therefore may go unnoticed or be dismissed as an idiosyncrasy of the child. The high adaptability of children has been well documented in various congenital limb malformations, for example in cases of radioulnar synostosis, with respect to forearm rotation [19,20]. However, as functional demands increase -through sports, learning musical instruments, or occupational activities - these limitations may nonetheless become clinically significant as the child becomes older.

Modern digital techniques, including three-dimensional analysis and preoperative planning of corrective osteotomies with patient-specific guides, are particularly useful in complex cases [21–23]. In fractures of the growing skeleton, joint surface geometry and soft tissue envelopes change with bone growth, adding further complexity. Nevertheless, corrective osteotomy remains a prerequisite for additional procedures, such as soft tissue release or ligament reconstruction. Notably, correction of alignment and joint orientation alone is often sufficient to restore wrist function and stability [24–26].

The use of free fat graft as an interposition material, as utilized in our revision surgery to minimize the risk of recurrent fibrosis, is predominantly applied in the operative treatment of congenital malformations, as described by Langenskiöld and Vickers [27,28]. Using this technique, re-ossification or excessive scarring following separation can be reliably avoided [29,30].

## Conclusion

In children with severely displaced distal forearm fractures, a potential Galeazzi injury should always be considered. Assessing DRUJ stability and congruence on initial trauma radiographs is often challenging. Therefore, explicit evaluation of the DRUJ during reduction under anesthesia or sedation is recommended. For cases managed conservatively, radiographic reassessment after 7–10 days is advised, when improved imaging projections are usually achievable.

Persistent or progressive motion limitations following pediatric forearm fractures are unusual and warrant further investigation. A persistent (sub)luxation can cause, in rare cases like the one we presented, progressive malformation of a child’s skeleton.

Modern techniques using three-dimensional analysis and preoperative surgical planning enable precise assessment and correction of complex deformities, allowing good to excellent functional outcomes even in complex deformities.
